# Dietary fiber in poultry nutrition and their effects on nutrient utilization, performance, gut health, and on the environment: a review

**DOI:** 10.1186/s40104-021-00576-0

**Published:** 2021-04-19

**Authors:** Rajesh Jha, Pravin Mishra

**Affiliations:** Department of Human Nutrition, Food and Animal Sciences, College of Tropical Agriculture and Human Resources, University of Hawaii at Manoa, Honolulu, HI 96822 USA

**Keywords:** Chicken, Dietary fiber, Environment, Gastrointestinal tract, Immunity, Intestinal microbiota

## Abstract

Dietary fiber (DF) was considered an antinutritional factor due to its adverse effects on feed intake and nutrient digestibility. However, with increasing evidence, scientists have found that DF has enormous impacts on the gastrointestinal tract (GIT) development, digestive physiology, including nutrient digestion, fermentation, and absorption processes of poultry. It may help maintain the small and large intestine’s integrity by strengthening mucosal structure and functions and increasing the population and diversity of commensal bacteria in the GIT. Increasing DF content benefits digestive physiology by stimulating GIT development and enzyme production. And the inclusion of fiber at a moderate level in diets also alters poultry growth performance. It improves gut health by modulating beneficial microbiota in the large intestine and enhancing immune functions. However, determining the source, type, form, and level of DF inclusion is of utmost importance to achieve the above-noted benefits. This paper critically reviews the available information on dietary fibers used in poultry and their effects on nutrient utilization, GIT development, gut health, and poultry performance. Understanding these functions will help develop nutrition programs using proper DF at an appropriate inclusion level that will ultimately lead to enhanced DF utilization, overall health, and improved poultry growth performance. Thus, this review will help researchers and industry identify the sources, type, form, and amount of DF to be used in poultry nutrition for healthy, cost-effective, and eco-friendly poultry production.

## Dietary fiber and their use in poultry nutrition

**Fig. 1:**
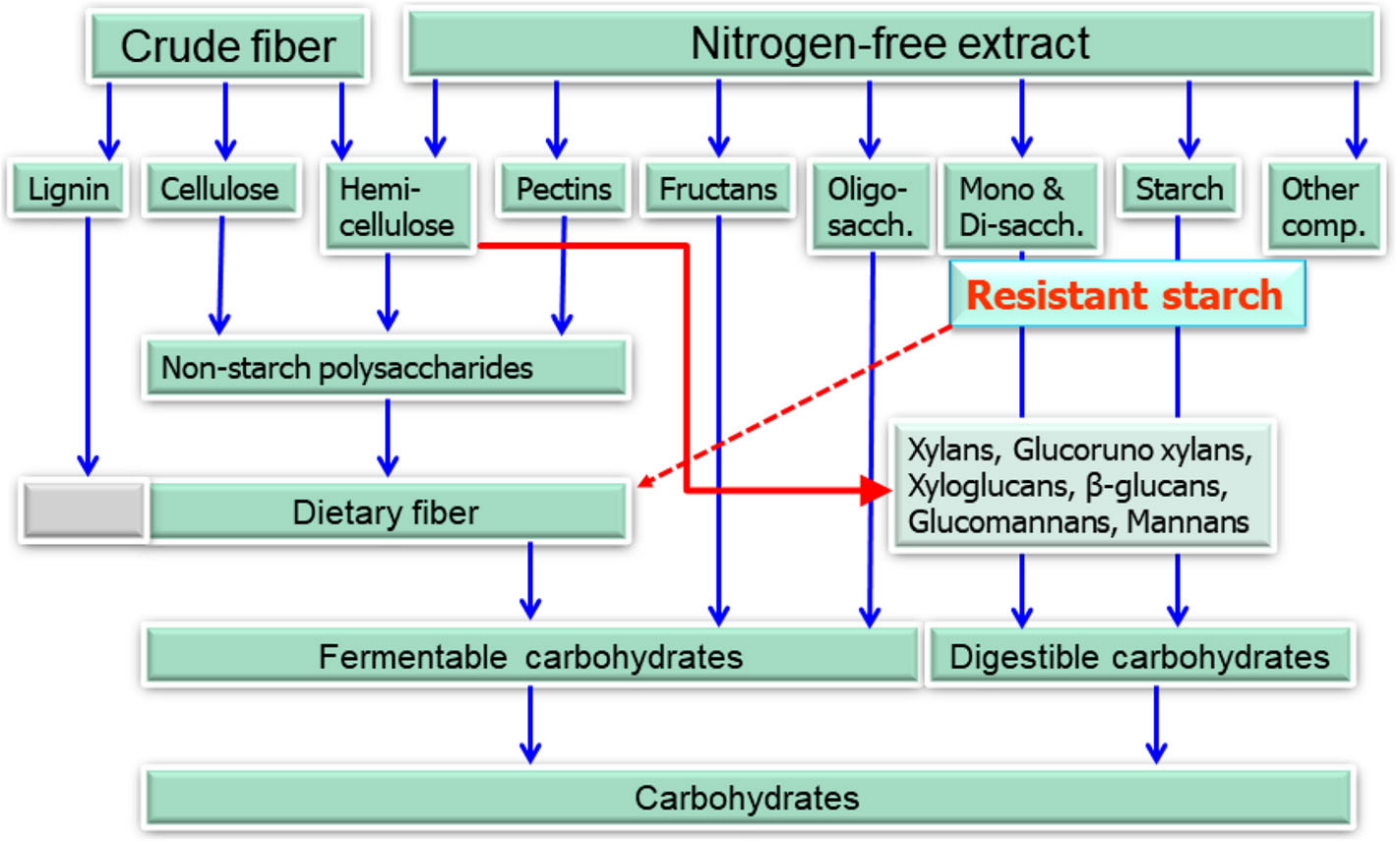
The major components of dietary fiber as part of the total carbohydrate

The term dietary fiber (DF) was first put forward by Hipsley [1] as a shorthand term for non-digestible constituents that make up the plant cell wall. However, the definition of DF has been continuously debated, without any universal agreement so far. The most accepted definition of DF is “all polysaccharides and lignin, which are not digested by the endogenous secretion of the human digestive tract,” and currently, most animal nutritionists are using either a physiological or a chemical definition [2, 3]. For the physiological definition, DF is “the dietary components resistant to degradation by mammalian enzymes”, while the chemical definition describes it as “the sum of non-starch polysaccharides (NSP) and lignin.” The major components of dietary fiber as part of the total carbohydrate are presented in Fig. 1.

It is common to characterize DF based on its solubility in water. The soluble fiber such as β-glucans from barley and oats, arabinoxylans from wheat and rye, pectins from fruits and sugar beet pulp (SBP) increases intestinal viscosity and decreases the rate of feed passage, which in turn reduce the feed intake (FI) and the rate of nutrient absorption and may have an influence on growth performance of poultry [4]. In contrast, insoluble fiber, which is rich in oat hulls and sunflower hulls (SFH), might have different effects on the GIT than soluble fiber [5, 6]. A moderate level of insoluble fiber in poultry diets may increase chyme retention time in the upper part of the GIT, stimulating gizzard development and endogenous enzyme production, improving the digestibility of starch, lipids, and other dietary components [7]. It has been agreed so far that soluble fiber is more rapidly fermented as compared with insoluble fiber [8]. However, this view is changing now as there is no standardized method for separating soluble and insoluble fiber. The soluble and insoluble fiber fractions may vary depending on temperature, water or buffer as the solvent, and fiber to solvent ratio, leading to significant limitations in the classification of fiber [9]. For details on dietary fibers’ basics, including definition, classification, characterization of methods, and physiological functions, readers are referred to Jha and Berrocoso [3].

Traditionally, DF has been considered as an antinutritional factor and a diluent in poultry diets. Several reports show a strong negative correlation between the fiber content of the diet and the digestibility of protein and fats. Those reports also indicate that increased fibrous components of the diet reduce growth performance and impair nutrient retention in turkeys and broiler chickens. These DF cannot be hydrolyzed by the digestive enzymes in the small intestine but can be fermented to a certain degree by the microflora in the GIT [6, 9]. The end-products of microbial fermentation are various gases (H_2_, CO_2_, CH_4_), lactic acid, and short-chain fatty acids (SCFA). Soluble NSP can be digested significantly in the cecum. In contrast, the insoluble NSP fraction remains almost entirely undigested, but when they are fermented to SCFA, the energy can be utilized by the host animals to a certain extent [10]. The addition of soluble NSP to a broiler chicken diet drastically increased SCFA production in the ileum, which was easily reversed when the NSP was depolymerized with an enzyme, and SCFA levels in the ileum were negatively correlated with apparent metabolizable energy and starch digestion. This is detrimental to the performance and well-being of poultry. However, it has been demonstrated that the inclusion of moderated amounts of different fiber sources in the diet has beneficial effects. The use of diets high in fiber, especially insoluble fiber, may reduce the incidence of cannibalism. Therefore, it may be used as an alternative to beak trimming in some production systems [11]. It can also improve poultry digestive organ development, especially gizzard activity, increase bile acids and enzyme secretion, and change intestinal microflora. These changes result in improved nutrient utilization, growth performance, and eventually, satiety and animal welfare [7]. In addition, fibers in poultry diets may positively affect gut health by preventing the adhesion of specific pathogen bacterial populations to the epithelial mucosa [12].

As mentioned above, fibers are found to have health benefits and can be used as an alternative to antibiotic growth promoters (AGP). Antibiotics were widely used in poultry feed to prevent, control, and treat diseases and infections. However, the indiscriminate use of antibiotics in the feed may result in residues in meat, selection of resistant bacterial species, and forming colonization of antibiotic-resistant bacteria also easily transferred to formally susceptible bacteria, which affects both animal and human health negatively. With the ban or strict regulation on the use of antibiotics in feeds as growth promoters in many jurisdictions globally, there has been an increased incidence of enteric disorders in poultry [13]. Therefore, researchers are working to identify alternatives to AGP, feeding highly digestible ingredients, enzyme supplementation, and using different feed processing techniques to improve the growth performance of birds in the post-antibiotic era. Feeding moderate amounts of fibers in diets has been considered as one of the alternatives proposed to improve nutrient digestibility and growth performance due to their role in the development of the GIT and to modify the characteristics of the intestinal contents. In addition to feed additives such as probiotics, prebiotics and plant extracts, feed ingredients or their components such as the inclusion of whole cereals, feeding coarse mash diets, and increasing the level of fiber in diets have been explored as nutritional strategies to reduce the incidence of GIT-related problems [7].

Poultry production has been growing rapidly during the past half-century throughout the world. Obtaining the most significant benefit from the least production cost is an essential issue to consider in the poultry industry currently. Corn, wheat, and soybean meal (SBM) are the primary components of poultry diets, which supply a significant portion of energy and protein. In addition to corn, wheat, and SBM, cereal grains and cereal coproducts rich in fiber can also be considered a substitution in monogastric animal diets [3]. The conventional sources of coproduct rich in fibers are wheat bran, SBP, SFH, sunflower meal (SFM), fuzzy cottonseeds, oat hulls, soybean hulls (SBH), pea hulls [7, 14–17]. Broiler chickens fed with 23 g SBP per kg diet had higher feed intake and body weights and better feed efficiency than control birds [15]. The use of cottonseed meal in the poultry diet is limited due to the presence of gossypol, cyclopropenoid fatty acids, poor protein quality, and high fiber. However, processing like solid substrate fermentation and extraction with organic solvents reduce free gossypol content and improve the nutritional value of cottonseed meal. Many workers reported the superiority of the combination of cottonseed meal with other protein supplements on body weight gain (BWG) and alleviated the depression in laying performance of poultry [16]. The dietary inclusion of coarse insoluble fiber sources like oat hulls in moderate amounts (between 2% and 3%) usually improves the growth performance of broilers fed low-fiber diets [7]. From the outcomes of three studies, Vierira et al. [14] concluded that substituting SBM with SFM as a primary protein source in laying hens has no adverse effect on egg weight, shell quality, mortality, egg production, and body weight. However, feeding the alfalfa to laying hens during molting may reduce the negative influence on egg production [18]. In recent years, plant extracts are being paid considerable attention as feed additives by virtue of their advantage of being natural and environment friendly. Plant extracts contain polysaccharides that possess immune-modulating effects and regulate the balance of the neuroendocrine-immune network. For e.g., adding alfalfa polysaccharides in the broiler diet enhanced immune function by improving the relative thymus, spleen, and bursa weight [19].

Different types of fiber differ in structure, solubility, water holding capacity, viscosity, bulking capacity, and other physiochemical properties. On the other hand, chemical composition and fermentation capability, as well as the grade of lignification of the source of fiber, may affect the diversity and population of the resident microbiota in the GIT [3]. Under practical conditions, factors like types of housing (cage vs. floor pens), composition and physical structure of the basal diet (i.e., type of cereal), type and level of inclusion of fiber and feed form (mash vs. pellet or crumbles) influence the utilization of fiber in poultry diet. Consequently, FI, GIT development, gut physiology and microbiota, growth performance, and immune system may vary depending on these factors [4, 7].

## Role of dietary fiber in GIT development

Despite being a monogastric animal, the GIT of poultry is different from pigs and humans. The GIT of poultry is notably different from other species as it is much shorter and lighter. However, it is relatively much longer (i.e., cm/kg body weight) and heavier (i.e., g/kg body weight) in poultry compared to other livestock. The GIT system’s function includes digestion, absorption, and protection, and the structure of the gut is well adapted to perform these functions. As the site of digestion, GIT maximizes nutrient utilization to reduce substrate for bacteria and support epithelial cell growth and differentiation. GIT also supports gut tissue integrity, prevent adhesion of pathogenic bacteria, balance microbial populations with low numbers of potentially pathogenic strains, support appropriate immune response, and control inflammation. The effective functioning of the GIT and its health are important factors in determining animal performance in growth, meat, and egg quality. Also, the development of the GIT is an essential aspect of growth, especially the development of functional digestive organs during the early post-hatching period of chicks [20].

Dietary fiber affects the length and weight of the GIT. There is also strong evidence that the differences in the weight of organs are highly related to differences in the type of fiber [10]. Diets with increasing levels of pea fiber decreased the dry matter (DM) in droppings and increased excreta output relative to DM intake. The digestibility of all nutrients also decreased with increasing fiber levels. Adaptation to increased inclusion of DF levels increases the size of the GIT, with pea fiber exerting a more substantial impact than wheat bran or oat bran. The length of the intestine, particularly the length and weight of the cecum, increased with the fiber level. These changes will impact energy metabolism as visceral organs have a high rate of energy expenditure relative to their size. Later, a study by Hetland et al. [21] indicated that performance does not decrease when an insoluble fiber is included in moderate levels to broilers or layers despite reduced nutrient concentration in the diet. The modern, highly selected birds also show their ability to compensate for reduced nutrient concentrations due to the insoluble fiber by increasing feed consumption. The increased bulk of ingesta due to insoluble fiber seems to be handled by a larger capacity of the digestive system and a faster passage through the GIT. In poultry, reduction in particle size increases digestive efficiency as a consequence of a greater interaction of the resulting larger surface of grains with the digestive enzymes in the GIT. However, the large particle size can promote GIT development, especially the gizzard function. When the gizzard is well-developed, an improvement in gut motility is also observed, which may reduce the risk of gut pathogens colonizing the lower segments of the GIT, thus reducing the risk of gut diseases, including salmonellosis and coccidiosis [22]. Diet rich in high fiber content may produce greater dilatation of proventriculus with the increase in size and its contents. The coarse fiber particles are selectively retained in the gizzard that ensures a complete grinding and a well-regulated feed flow and secretion of digestive juices [4]. Jiménez-Moreno and Mateos [4] proved that the inclusion of 3% SBP or oat hulls increased gizzard weight in broilers fed similar types of diets. The inclusion of oat hulls or SBP increased to 5% in 36 days old broilers increased the gizzard weight and its contents, and reduced gizzard pH. An accumulation of oat hull particles stimulates the gizzard’s grinding activity, allowing for the better development of the muscular layers and causing an increase in organ size. Compared with oat hulls, the effects of fiber inclusion on the enlargement of GIT were more evident with SBP, which may relate to the higher pectin content of SBP. Soluble fiber particles such as those from SBP, retain water and swell in the digestive tract, causing digesta bulk and physical distension of the walls and increase in size. Kimiaeitalab et al. [23] reported that all the organs of the GIT were heavier and the small intestine and cecum were longer in broilers than in pullets when feeding with same high fiber containing SFH meal compared to SBM, consistent with the greater GIT capacity and average daily FI of broilers. An increase in the weight of GIT is important to pancreatic enzymes secretion to achieve better growth in birds during the early stage of life.

Insoluble fibers comprise a large proportion of the endosperm cell walls, which physically limit access of digestive enzymes to the nutrients within the cell. In contrast, the soluble fibers tend to result in viscous conditions in the digestive tract, which can adversely affect digestion and nutrient absorption. After escaping the small intestine, both soluble and insoluble DF are fermented by the microflora in the large intestine, accompanied by an increase in SCFA, resulting in a decrease in intestinal pH [3, 22]. Considering the prebiotic effect of DF, the stimulation of beneficial bacteria such as *Lactobacillus* can optimize gastrointestinal health as the lactobacilli’s attachment to the intestinal mucosa can prevent the pathogen growth in the distal part of the GIT and protect animals from GIT infection [3]. The reduction of the pH of gizzard content by the inclusion of fiber probably results from higher hydrochloric acid (HCl) secretion from the proventriculus, resulting from the longer retention time of the digesta in the gizzard. However, the inclusion of fiber did not affect the pH of the duodenum. More research needs to examine the effect of increasing the fiber content of the diet on intestinal digesta in birds [4].

Dietary fiber may influence epithelial morphology, which depends on the characteristics of the DF, the level of inclusion, the age of the bird, and the site in the intestinal tract. An excess of DF added to the diet (7.5% of pea hulls or SBP) increased the abrasion of the mucosal surface of the small intestine, shortened the villus, and increased mucus output [24]. This resulted in reduced absorptive villus surface and hindered nutrient retention. Moreover, the highest villus height to crypt depth ratio was observed with 2.5% pea hulls inclusion in the diet. Similar conclusions were made by Kimiaeitalab et al. [23] that villus height and crypt depth were higher in broilers than in pullets at 21 d of age when fed with the same SFH meal, which was consistent with the greater GIT capacity and average daily FI of broilers. Tüzün et al. [25] reported that increasing dietary crude fiber content in Nick Chick pullet from 30 to 40–45 g SFM/kg level increased VH, villus weight, the VH/CD ratio and surface area. Similar results were also reported by Koçer et al. [26] with the inclusion of 47 g SFM/kg diet, but decreased with increased level of SFM (97 g SFM/kg) in Nick Chick white laying hens. A high villus height to crypt depth ratio is considered as an indicator of better maturity and function of the intestinal mucosa. Thus, it is essential to determine the optimal concentration and source of DF to support better GIT development.

## Effects of dietary fiber on nutrient utilization

Utilization of nutrients may vary on the components of DF present in the supplied feed as well as the function of the gut. For e.g., DF present in wheat, corn, and rye are rich in the NSP arabinoxylan, while oats and barley have much higher levels of highly soluble β-glucan and SBM has a relatively high β-mannan content [27]. The composition of the NSP portion of a feedstuff will determine how it behaves once ingested. The fiber’s solubility and water holding capacity determine its viscosity, and fermentability impacts lower gut utilization and health. High viscosity will decrease the rate of endogenous enzyme diffusion into the digesta, which will reduce nutrient digestion. Additionally, highly viscous digesta will have less interaction with the brush border membrane enzymes, which also decreases digestibility and nutrient utilization [28, 29].

In the study of Sadeghi et al. [30], chickens fed with SBP (30 g/kg) and SBP/rice hull (15 g/kg each) supplemented diets showed lower daily weight gain at the age of d 14–28. This might be because of the increased viscosity of digesta due to the soluble DF pectin found in the SBP and results in lower enzyme diffusion into the digesta and decreased interaction of the digesta with the intestinal tract surface. Additionally, the researchers also found that SBP feed chickens had shorter villi height in the duodenum and ileum, indicating reduced nutrient absorptive capacity. These effects can be offset by supplementing exogenous enzymes, which increase the digestibility of high DF feedstuffs. The primary two ingredients of poultry diets on a global scale are corn and SBM. SBM contains moderately high levels of β-mannan/β-galactomannan compared to the total NSP content of the feedstuff. Monogastric animals cannot cleave the β-1,4 bonds present in this DF, leading to lower nutrient digestibility. There are higher levels of β-mannan found in non-dehulled SBM, as β-mannan resides in hulls [27, 31]. Numerous studies have shown the efficacy of supplemented β-mannanase enzymes to offset known deleterious effects of β-mannan on weight gain, feed conversion, and overall animal performance [27, 32, 33]. Singh et al. [33] looked at NSP utilization in both low and high fiber diets while supplemented with protease, amylase, xylanase, and three species of *Bacillus* as probiotics. They found that the treatments increased nutrient utilization regardless of DF level. Another study by Li et al. [34] showed that the low-fiber diets underutilized energy and crude protein compared with higher fiber diets. Later, they also reported that low fiber diets impacted the cecal microbiota of birds by decreasing microbiota diversity and relative abundance [35].

Enhanced organ development and functionality can result in increased nutrient digestibility within the GIT. DF has been seen to have a positive effect on gizzard development [36]. The well-developed gizzard is linked with improvements of the digestive organs’ mucosal surface within the GIT [37], leading to improved nutrient digestibility and absorption. Abdollahi et al. [38] also reported that the feeding diets supplemented finely ground oat hulls (30 g/kg) or wood shavings (30 g/kg) increased gizzard weight over feeding whole wheat at the same inclusion rate and improved the nutrient digestibility. This was influenced by the type of NSP present in the dietary treatments as opposed to overall particle size. While this improvement is seen with diets containing structural components such as fiber, the type of DF sources determines the rate of effectiveness. Insoluble fibers have a more extensive effect on gizzard functionality when compared with more soluble fibers. This improves the gizzard function [39] and stimulates HCl production in the proventriculus via mechanoreceptors [40]. This will lead to a low pH in the upper part of the GIT, which will favor pepsin activity and facilitate the mineral salts’ solubility and absorption [41]. Therefore, the inclusion of insoluble fibers might benefit the nutrient digestibility in poultry [42]. This was reported by González-Alvarado et al. [5], where oat hulls were compared with SBP. The oat hull diet containing more insoluble fiber enhanced gizzard functionality when compared with the diet containing SBP. Similar results were found earlier in the study of González-Alvarado et al. [43] and Jiménez-Moreno et al. [44] when corn and rice were supplemented with oat hulls and soy hulls with or without heat processing of the diets. The heat processing changes the texture and compounds of starches and increases the digestibility [45]. They found that the rice feeding improved the total tract apparent retention of nutrients and feed conversion of the birds during the starter phase. The fiber in the diet was found to increase the gizzard weight and activity, which is evident in better diet digestion and nutrient utilization. Ling et al. [46] found that the DF levels affected the development of the upper GIT of the goslings when grit was supplemented to improve the gizzard activity and also improved digestion and nutrient absorption.

Poultry fed various amounts of DF have been shown to improve the utilization of most nutrients found in the feed provided [47, 48]. Amerah et al. [48] looked at how the starch digestibility was affected when using a wheat-SBM diet that included 6% wood shavings. The ileal digestibility of starch increased from 98.5% to 99.4% due to the increased DF content. High fiber SFM has both positive and negative effects on the digestibility of nutrients. A corn-based pelleted diet containing 30% high fiber SFM increased apparent ileal digestibility of crude protein and fat but decreased dry matter and energy digestibility [47]. While starch may be effective in increasing protein and fat digestion, the SFM impact negatively on nutrient utilization.

Prebiotic fibers can be used not only to improve growth performance but also to increase nutrient utilization. Houshmand et al. [49] investigated the ability of prebiotics to compensate for calcium (Ca) deficiency in poultry diets. He fed birds with a low Ca control diet and a prebiotic supplemented diet and found that the prebiotic supplemented group had no deficiency effects of low Ca in diets. Tako et al. [50] found that the prebiotic extracts from wheat grains were able to increase the beneficial microorganisms in poultry gut and iron absorption in both *in vitro* and *in vivo* conditions. The increase of Bifidobacteria and Lactobacilli with the presence of prebiotics might have an influence on iron bioavailability for the long term. Therefore, prebiotics might help in avoiding micronutrient malnutrition. These studies reveal the advantages of prebiotic fibers as dietary supplements to improve poultry growth performance and nutrient utilization. Also, mannanoligosaccharides extracted from *Saccharomyces* spp. of yeast outer cell wall was found to maintain gut health and increase the villi lengths [51]. Their results showed an increase in villi uniformity and integrity, which all contribute to increased nutrient absorption. Many positive effects of the use of prebiotic fibers on the intestinal mucosa have been reported, among which a significant increase in villus height was observed in three segments of the small intestine of birds [52]. Loddi [53] reported longer villi in the duodenum of birds fed with soluble fibers at the age of d 7 and 21. Similarly, Pelicano et al. [54] observed longer villi lengths when birds were fed with soluble fibers having mannanoligosaccharides. Aside from the two types of fiber (soluble and insoluble), Singh et al. [33] conducted a study on the different levels of fibers (low and high) in combination with enzymes (xylanase, amylase, and protease) and probiotics and found that supplementing additives help in increased nutrient digestibility even in high fiber diet. However, the supplementation of fibers in poultry diets does not always benefit growth performance and nutrient utilization. In the experiments conducted by Sadeghi et al. [30], villi lengths were found to be reduced in the SBP and rice hulls supplemented groups of birds which cause a reduction of nutrient absorption in the jejunum, thereby increased excretion of useful absorbable nutrients. Another study by Sadeghi et al. [55] reported that supplementation of basal diet addition with rice hull (40 g/kg) and SBH (40 g/kg) could ameliorate adverse effects of coccidiosis on duodenal villus height but addition of only SBH in basal diet has positive response only on villus height to crypt depth ratio. Similarly, inclusion of wheat bran at a dose of 30 g/kg increased the villus height and villus height to crypt depth ratio [56].

## Effects of dietary fiber on performance

High fiber diets usually mean relatively low energy density that may decrease FI, feed conversion ratio (FCR), and BWG of poultry. Pettersson and Razdan [15] observed that FI in 18 d-old chicks was reduced when the level of SBP of the diet was increased from 2.3% to 9.2%. Similarly, Jiménez-Moreno et al. [24] reported that an increase in the level of the fiber sources from 2.5% to 7.5% linearly reduced average daily weight gain from 1 to 12 d. However, the inclusion of a fiber source in the diet tended to reduce FI of the broiler for the first 12 d of age, but the effect disappeared thereafter. These studies’ discrepancies might be due to the soluble fiber content in SBP, especially high pectin content and its high water holding capacity and swelling capacity. These physicochemical characteristics result in an increase in digesta viscosity and a longer retention time of the digest in the GIT, eventually affecting voluntary FI. Considering the physiology of insoluble fiber, the inclusion of moderate amounts of insoluble DF should not affect voluntary FI. González-Alvarado et al. [43] studied the effects of the inclusion of 3% oat hulls or soy hull into a corn-based control diet that contained 2.5% crude fiber or a rice-based control diet that had 1.5% crude fiber. The inclusion of hulls reduced FI and improved FCR but did not affect BWG initially (from d 1 to 4). From 14 to 21 d of age, chicks fed hulls had higher BWG and FI and better FCR than chicks fed the control diets. Consequently, the inclusion of hulls improved BWG and FCR without affecting the FI. Probably, the level and type of DF, as well as the age of the bird, modifies the response of poultry concerning FI and FCR. Mateos et al. [7] suggested that young birds’ response to additional insoluble DF depends on the ingredient composition of the control diet, with effects being more pronounced when it is low in fiber. For example, González-Alvarado et al. [5] reported that the dietary inclusion of 3% SBP, a source of soluble DF, reduced FI from 25 to 42 d of age as compared with a diet containing 3% oat hulls. However, no adverse effects of SBP inclusion were observed during the first 10 d of life. In the study of Kimiaeitalab et al. [23], the increased inclusion of sunflower hull diet did not affect FI in both pullets and broilers from 0 to 21 d of age, which shows consistency with data of Walugembe et al. [57]. Walugembe et al. [57] also observed the non-significant decrease in feed efficiency in broiler chicks fed higher fiber diets and a non-significant increase in feed efficiency in layer chicks fed higher fiber diets. In contrast, Saadatmand et al. [58] observed a decrease in feed intake and weight gain significantly when fed with 30 g/kg SBP and rice hull. Overall, the mortality of poultry was not related to the dietary treatments containing different levels of fiber [23, 57]. Vierira et al. [14] reported that substituting SBM with SFM has no adverse effect on egg weight, shell quality, and egg production. Similarly, Hartini et al. [11] found that diet with different types of fibers (insoluble fiber and soluble fiber) did not affect egg production. Also, Roberts et al. [59] reported that high fiber feed ingredients of laying hen’s diet did not affect egg production or N balance negatively but decreased NH_3_ emission from manure, a positive indicator for the environment. However, one treatment group containing corn as the fiber source showed a significantly different egg yolk color. It may be due to a high content of xanthophylls in corn. It provides light to examine the effects of the different fiber sources on egg production and yolk color.

## Effects of dietary fiber on gut microbiota

It is understood that the development of the gut microbiota starts at the time of hatch; bacteria can be obtained from the environment, the mother, the feed, and the farm workers that touch the chicks at post-hatch. Moreover, these bacteria are colonized quickly within 24 h while the ileum and cecum get dominated after one-day post-hatch [60]. The number of bacteria in the small and large intestine will increase ten folds after three days. The adult small intestinal and cecal microbiota of chicken are entirely developed within one month. But, time for the establishment of stable gut microbiota can be reduced by optimizing the breeding conditions and feed quality [61]. Thus, studies have looked at modulating the gut microbiota by nutrition programming in poultry, both the pre-hatch and post-hatch period of life [20]. Zhang et al. [62] fed different doses of chitooligosaccharide (COS) and chlorella polysaccharide *in ovo* and found that COS supplementation (5 mg/egg) increased the beneficial bacteria population compared to the control group.

Gut microbiota consumes around 20% of the dietary energy, and it can be considered a highly metabolic organ. It mainly includes bacteria, fungi, protozoa, and viruses. However, there is still a large number of bacteria in the gut that are unknown and unclassified. Increasing evidence prove that around 700 species of microbiota colonize, including beneficial bacteria; *Lactobacillus*, *Bifidobacterium*, *Enterococcus* species and detrimental bacteria; *Clostridium* species [63, 64] in the GIT of poultry, and their abundance and diversity vary among the different region of GIT. Obviously, low tolerable conditions and fast digesta passage regions usually have less microbiota. Gut microbiota promotes enzyme secretion, contributes to the process of digestion and absorption. It also regulates energy metabolism, prevents mucosa infections, and modulates the immune system [65]. Therefore, maintain the host homeostasis and aids in the production of SCFA [66]. Moreover, some byproducts of microbiota metabolites can stimulate the neuroendocrine cell, which plays an important role in the development of poultry [22].

Fiber levels in diets may modify the growth and composition of the microbiota. Many soluble fibers function as prebiotics when in feedstuffs, directly promoting the growth of beneficial gut bacteria and production of SCFA [30, 60, 67, 68]. Similarly, insoluble fibers also act as a general nutrient diluent, which can be both helpful and harmful, potentially impacting the colonization of beneficial gut microbes [69]. Examination of bacterial cultures from the ceca of turkeys fed either a high or low fiber diet indicated that direct counts of microbes were significantly higher in high-fiber fed than low-fiber fed turkeys [70]. Turkey fed the high-fiber diet had a significantly higher number of *Peptostreptococcus* and facultative microorganisms in the cecum, while the number of *Escherichia coli* was significantly higher in low-fiber fed turkeys. Similarly, Xu et al. [71] observed that the number of *Bifidobacterium* and *Lactobacillus* significantly increased, while the number of *Escherichia coli* significantly decreased in GIT when broilers were fed with fructooligosaccharide as compared to control. A similar result was also reported by Chen et al. [72] when fed with an oligosaccharide.

Moreover, supplementation of fructooligosaccharide in chicken diets improved amylase and protease activities in the small intestine, proving that colonization of beneficial gut microbiota stimulates the intestinal digestive enzyme activities [71]. Similarly, the study of Abazari et al. [73] also showed that adding rice husk as a lignocellulose source could promote the growth of beneficial *Lactobacillus* bacteria and reduce the population of pathogenic bacteria such as some *Escherichia coli* in the ileum and cecum of broilers at 42 d of age while fed with 7.5 g/kg and 15 g/kg of feed with a particle size of 1-2 mm and below 1 mm. The combination of mannan-oligosaccharide and β-glucans, in the form of whole yeast, has also been shown to increase *Lactobacillus* bacteria in the gut [74].

Jørgensen et al. [10] observed the extent of DF degradation by microbial fermentation closely relates to the H_2_ and SCFA (mainly lactic acid and acetic acid) excretion. They found higher microbial fermentation with increasing NSP level, while insoluble fiber was poorly fermented. This all information suggests a poor relationship between insoluble fiber content and the composition and quantity of gut microbiota. However, an excessive soluble fiber may cause some adverse effects in the gut. As one of the essential substrates for bacterial fermentation, DF modulates the balance between gut microbiota and gut mucosa, including mucus layer, digestive epithelium, and gut-associated lymphoid tissues. As mentioned above, the physical form of DF affects the morphological and physiological characteristics of the intestinal tract, which may also affect the gut microbiota [75].

Wu et al. [76] showed that high dietary soluble fiber (7% of soy hull) inclusion in the broiler diets significantly increased acetic, propionic, isobutyric and butyric acid, lactic, and succinic acid in cecal contents and depressed formic acid production compared with low dietary fiber when challenged with *Clostridium perfringens*. The study of Walugembe et al. [77] reported that the increase of DF decreases butyric acid without bringing any change in other SCFA concentrations, while propionic acid and acetic acids were not varied with the feeding of high or low DF. The analysis of the cecal microbiome using cladograms from WGS depicted that there was an increase in the relative abundance of the orders Selenomonadales, Enterobacteriales, and Campylobacterales in the cecal sample from the broilers fed with a high fiber diet than the cecal samples collected from the broilers fed the low fiber diet. The increase in SCFA reduced the abundance of *Faecalibacterium* genus. The low fiber diet decreased the abundance of *Bacteriodes* genus in both broiler and laying hens. But an increase in abundance of *Escherichia coli* and the *Campylobacter* genus occurred in the birds fed high fiber diets. Soluble fiber (barley β-glucans or wheat arabinoxylans) in diet shows a negative effect on the growth. It has also proved to favor the expansion of potential pathogens, *Escherichia coli*, *Clostridium perfringens,* and sometimes the villi can be atrophied because of the impact of the soluble fiber in the intestine for a long time [78].

One of the main components that affect the presence of the gut microbiota is the chemical composition of the digestive system itself. Beneficial microbes desire a low pH environment, and fluctuations or nutrient deficiencies can lead to colonization by detrimental microbes, and DF has been seen to have an impact on the chemical composition of the gut [79]. *Lactobacillus* species are particularly beneficial in maintaining the pH of the digestive tract, as they produce lactic and acetic acids, which help to lower the overall pH [67]. Low to moderate amounts of resistant starches have been shown to positively impact the production of HCl, bile acids, and various enzyme secretions by the digestive tract, which helps in the growth and maintenance of the gut microbiota [7]. The acetic acid, in particular, is a common and useful SCFA that is produced by *Lactobacillus* species, which are found in multiple regions of the digestive tract acts effectively in the reduction of populations of members of the *Campylobacter* genera, which are known to cause gastroenteritis, leading to diarrhea and dehydration [67, 80].

As with many of the previously described benefits of fiber in poultry diets, the quantity present in the feedstuffs has a measurable effect, with high amounts of fiber, resulting in potentially harmful effects. While low levels have provided increases in bacteria efficient in degrading polysaccharides into SCFAs, such as *Helicobacter pullorum* or *Megamonas hypermegale*, high levels have resulted in detrimental populations of pathogenic Selenomonadales and Enterobacteriales establishing colonies and proliferating in poultry digestive systems, particularly within the ceca [77]. High fiber diets have also seen increases in *Escherichia coli* and *Campylobacter jejuni*, which can cause gut inflammation and deteriorate the overall health of the host [77, 81].

## Effects of dietary fiber on the immune system

Poultry has both innate and acquired immune systems, and both are highly efficient in defenses. Unfortunately, neonatal poultry exhibits a transient susceptibility to infectious diseases during the first week of life. Because of this deficiency in the functional ontogeny of the avian innate and acquired immune defenses, and with the continued threat of exotic and emerging diseases and concern over the use of AGP in poultry production, there is a severe and urgent need to find safe and practical alternatives to prevent or control pathogens. Thus, more studies are needed to develop an alternative and appropriate nutritional program, considering the interaction between dietary bioactive food components and the immune response to reduce susceptibility to infectious disease [82]. Appropriate nutrition programs may help minimize the incidence of diseases and enhance immunity by increasing relative immune organ weight, improving relative immune gene expression, and promoting the generation of antibodies in the blood [83]. Theoretically, such measures would be easier and more economical to introduce to the poultry industry because of the genetically homogenous populations of domestic fowl in the commercial environment. It is also feasible to have several feeds available, each with its own immune-modulating nutrient to direct the immune response in a specific direction [84]. DF can be used as a cost-effective nutrient to modulate the poultry immune system. With its high fiber content, Alfalfa has been shown to have a very long transit time in the GIT of chickens. This increase in transit time favors bacterial degradation of DF into fermentable substrates such as fructo-oligosaccharides that can later be utilized by microbes to produce SCFA. Increasing the fiber content in a diet benefits the digestive system by normalizing colonic function and increasing fecal weights and evacuation frequency. These actions help to maintain the small and large intestine functions by increasing mucosal structure and function and increasing the populations of commensal bacteria in the GIT. Adding polysavone, a natural extract from alfalfa, to male commercial broiler birds diet improved the relative thymus and spleen weights at 6 weeks of age and the bursa weights at 4 and 5 weeks of age. The proliferation of T and B lymphocytes with polysavone treatment was significantly greater than the control group when birds were 4 and 6 weeks of age. The inclusion of polysavone in the diet also resulted in a significant increase in serum anti-Newcastle disease virus hemagglutination inhibition antibody titer. This experiment showed that adding polysavone may enhance immunity without any adverse effect on the performance of broiler chickens [19]. Goblet cells in the GIT produce mucin, which can help to improve the gut barrier as pathogenic microbes cannot penetrate through the dense mucous layer. Arabinoxylan from wheat bran has been found to increase the number of goblet cells, which secretes not only mucin but also protein barrier factors, hence protecting intestinal epithelial cells. Similarly, algae-derived polysaccharides supplementation enhanced antioxidant capacity and intestinal barrier functions in broiler chickens [85]. Dietary pectin inclusion upregulated the IL-12 expression in the ileal mucosa to increase interferon-γ production in cecal tonsils and decreased the invasion of sporozoides in ileal enterocytes during infection with *Eimeria maxima*. Also, it provided some benefits in the face of an active parasitic infection when the diet was supplemented with pectin [82]. Pectin may decrease the ability of the pathogen to colonize within the GIT through physical exclusion that increases the digesta viscosity to protect the intestinal mucus layer from parasitic infection.

Cox et al. [86] studied the effects of β-glucans from yeast on the immune system of healthy, unchallenged chicks from hatch to d 14. Body weight gain, immune organ weight, and white blood cell ratios were unchanged between treatments, but the cytokine to chemokine ratio, particularly between interleukin (IL)-8, interferon-γ, IL-13, and IL-4, were altered in birds receiving the β-glucan supplemented diet. The balance in ratio concludes that β-glucans affect some component of the immune system without negatively impacting chick performance during good health. But when challenged with *Eimeria* species with yeast-derived β-glucan, it also alters the cytokines profile of chickens. Helper T cell activity, part of the cell-mediated adaptive immunity, was increased, and compounds related to the inflammatory response, part of the innate immune system, were upregulated [87]. While Tian et al. [88] supplemented yeast-derived β-glucans in *Eimeria* and *Clostridium perfringens* challenged chicks where he found improved gut morphology and integrity, decreased *Clostridium perfringens* populations, and increased antibodies against the infecting bacteria and parasites. The effects seen with β-glucans are structural dependent. Ott et al. [89] concluded that the highly branched form of β-glucan is generally regarded as the form with the most potent immunostimulatory effects supplemented *Eimeria* challenged broiler chicks with linear, low branching β-glucans derived from algae.

β-Glucans also appear to play a role in intestinal barrier integrity during *Salmonella *Typhimurium infections. Chicks challenged with *Salmonella* Typhimurium and fed a diet supplemented with 1,3/1,6-β-*D*-glucans had increase Immunoglobulin A secretions into the jejunum in addition to better intestinal barrier integrity when compared to the challenged chicks without β-glucan supplementation [90]. β-Glucan supplementation may also be beneficial to increase the immune response after vaccination. Horst et al. [91] looked at the effects of β-1,3-glucan on Newcastle disease virus (NDV), avian infectious bronchitis virus (IBV), and infectious bursal disease (IBD) vaccine response over the course of 42 d. Antibody titers for NDV and IBV were significantly higher in β-glucan supplemented birds, but no difference was seen in IBD titers. Berrocoso et al. [92] fed the developing embryo with raffinose and found that *in ovo* supplementation increased not only the villus height and villus height:crypt depth ratio but also the expression levels of *CD3* and *chB6* genes, which are T cell and B cell marker genes, respectively.

When fermented by intestinal microflora, some soluble DF results in SCFAs i.e., acetate, propionate, and butyrate; butyrate has the immune-modulatory response in poultry [93]. Butyric acid is produced in the gastrointestinal tract by anaerobic bacterial fermentation, which has beneficial health effects in poultry [94]. To understand the immunomodulating effects of butyrate on the avian macrophage, Zhou et al. [94] conducted a study in which they treated a naturally transformed line of chicken macrophage cells named HTC with Na-butyrate in the absence or presence of *Salmonella typhimurium* lipopolysaccharide (LPS) or phorbol-12-myristate-13-acetate (PMA), a metabolic activator, and evaluated its various functional parameters. The results demonstrate that butyrate inhibited nitric oxide production, and reduced the expression of cytokines such as IL-6, IL-10 in LPS-stimulated cells, and enhance intestinal epithelial cell barrier function, the first line of defense against invading pathogens [93] and also helps in maintaining the physical barrier by stimulating goblet cell differentiation and mucus production. They help in maintaining gut health by promoting the function of colonic regulatory T cells by inhibiting the effector T-cells [95]. This action contributes to preventing excessive inflammation and thus shows the immunomodulatory property in the presence of agents that incite the cells; therefore, it has the potential to control inflammation and restore immune homeostasis.

Yeast cell wall, a mixture of β-glucans and mannan oligosaccharide, is another compound that is being investigated for immunostimulatory effects as an alternative to in-feed antibiotics [96]. In an experiment by Gao et al. [97], when broiler chicks vaccinated with an NDV vaccine and fed a corn-soybean based diet supplemented with 2.5 g/kg yeast cell wall showed higher antibody titers, serum lysozyme activity, better gut morphology, higher IgM concentrations, and IgA concentrations secreted into the small intestine than the control chicks. Alizadeh et al. [98] compared gene expression of immune factors (Toll-like receptors 2b, 4, and 21; macrophage mannose receptor; IL-12, IL-10, IL-4, IL-6, IL-18; and interferon-γ) between lipopolysaccharide (LPS) challenged chicks. Chicks fed a yeast cell wall supplemented diet had increased IgA levels compared to the antibiotic supplemented diet; IgG and IgM were unaffected by all dietary treatments, but the yeast cell wall diets had upregulated cytokine, Toll-like receptor 21, and macrophage mannose receptor expression. This differed slightly from Gao et al. [97] as IgM concentrations did not increase, but this could be due to the pathogens challenge while Munyaka et al. [99] saw a downregulation of IL12p35, Toll-like receptor 4, and IL-10 in the intestines of yeast cell wall supplemented unchallenged chicks compared to the control.

Li et al. [100] saw a decrease of *Clostridium perfringens* serum endotoxin when chicks were fed yeast cell wall supplemented feed, and there was an upregulation in cytokine mRNA expression. This is similar to the results found when isolated β-glucans were supplemented [88]. Ahiwe et al. [96] also saw positive results with yeast cell wall when fed to chicks challenged with subclinical necrotic enteritis from *Clostridium perfringens* and *Eimeria* coinfection. A yeast cell wall supplemented diet was fed from hatch to d 35 and challenged with *Eimeria* at d 9.

Yeast cell wall can also be beneficial against viral challenges. Awadin et al. [101] supplemented Fetomune Plus, a yeast plus vitamin supplement, with vitamin E in avian influence (H9N2) challenged chicks, and treated chicks had upregulated immune gene expression, higher body weights, and lower mortality rates than the control group.

Like broiler and layer, yeast cell wall supplement fed has effects in turkey. Huff et al. [102] fed turkeys either a commercial diet or the commercial diet supplemented with a yeast extract (Alphamune™ G; minimum 24% β-glucans and 5–10% mannan-oligosaccharide). Birds were challenged at 16 weeks of age with an unspecified bacterial challenge, and at 18 weeks of age were either stressed or not stressed (by either transportation or immunosuppression). Oxidative burst activity, an important event that occurs during phagocytosis of pathogens, was stimulated by the yeast extract in the unstressed birds. Still, in the stressed birds, there was no significant difference from the control birds. It is also reported that the heterophil to lymphocyte ratio, a measure of stress, was increased in all birds fed yeast extract, which led them to hypothesize that the increased immune activity seen may be because the yeast extract was a stressor itself. Again, Huff et al. [103] fed turkey either a yeast culture supplement continuously or intermittently from hatch to16 weeks. The birds were challenged with transportation stress and *Escherichia coli* at 6 and 12 weeks to stimulate the industry practice of moving between 3 houses throughout the grow-out period. The intermittently supplemented diet, given for 1 week after the stress event, improved the feed conversion ratio significantly in those birds and had lower *Salmonella* populations in the ceca; the continuously fed birds had lower *Salmonella* and *Campylobacter* populations in the ceca. The authors suggested that intermittently supplementing yeast may be better to decrease the effects of transport stress in growing turkeys.

Arabinoxylan, a water-soluble NSP found in high concentrations in wheat, has antinutritive effects, such as being a pro-inflammatory agent and increasing digesta viscosity, but research has shown derivatives of these compounds with appropriate source and amount may have beneficial effects [104, 105]. Yacoubi et al. [106] isolated arabinoxylans from wheat and added them to a broiler chick diet with a multienzyme, which created short-chain arabinoxylan polysaccharides. Birds fed this diet had increased production of cecal SCFA. In particular, butyrate presence increased significantly, which has been shown to have anti-inflammatory effects and inhibit pathogenic bacterial colonization in the intestine. Similarly, arabinoxylooligosaccharides promote bifidobacterial growth in the cecum of chickens and aids in maintaining gut health [107], which is beneficial during *Salmonella* Enteritidis challenge. Infected birds fed a diet supplemented with either 0.4% or 0.2% arabinoxylooligosaccharides from wheat bran show decreased *Salmonella* populations in the ceca and spleen [108]. Akhtar et al. [109] fed birds barley-derived arabinoxylans (200 mg/kg body weight or 300 mg/kg body weight) on d 7, 8, and 9 and then measured lymphocyte concentrations. Birds fed arabinoxylans enhanced macrophage activity, which the authors speculated was due to increased T-cell mitogens. Chitosan oligosaccharides (COS) are extracted from chitin, and supplementation of COS could increase the weight of the immune organs (bursa of Fabricius and thymus), increase IgG, IgA, and IgM and elevate antibody titers against Newcastle disease vaccines [110]. The study of Park et al. [111] reported that galacto-oligosaccharides (GOS) supplementation resulted in an increased number of *Alistipes* genus, *Lactobacillus intestinalis*, and *Faecalibacterium prausnitzii* in the ceca of broilers. Unlike other polysaccharides, GOS could not increase the count of *Bifidobacteria* and *Lactobacillus* in the ceca [112].

Astragalus polysaccharides (APS) is considered as a biological response modifier [113]. This polysaccharide can initiate the secretion of cytokines [114], activate T and B lymphocytes [115], and components of complement [116]. APS have immunostimulatory effects when the chickens are fed on this compound. This compound increases the proliferation of lymphocytes, T-lymphocytes percentage, antibody titers, relative weights, histology of immune organs, cytokine concentrations, and intestinal microbiota [113, 117]. Li et al. [113] also investigated the synergistic effects of probiotics and APS on immunity and gut microbiota in chickens. The result showed that antibody titer, peripheral blood acid α-naphthyl acetate esterase-positive T-lymphocyte percentage, relative weights and histology of immune organs, and microbiota of the birds had been changed positively. Shan et al. [118] studied the effect of oral administration of APS on jejunum mucosal immunity in chickens vaccinated against Newcastle disease, which resulted in higher VH:CD ratios (jejunal villus height-crypt depth ratio), increased IgA^+^ cells, and higher ND virus specific secretory IgA levels in jejunal contents [118]. Guo et al. [119] also studied *Eimeria tenella*-infected chickens by feeding polysaccharide extracts from 2 mushrooms (*Lentinus edodes* and *Tremella fuciformis*) and *Astraglaus membranaceus* to measure the cellular and humoral immune response of the chickens. Chickens from *Astragalus*-fed group produced the highest IgG at the initial stage of coccidiosis. But later, the mushroom-fed groups showed higher IgM and IgG levels than *Astagalus*-fed group. The feeding of polysaccharides of three types improved the overall mean of erythrocyte rosette-forming cells and erythrocyte-antibody-complement cells. Wang et al. [120] tested the effect of *Paulownia fortunei* flower polysaccharide (PFFPS) on cellular and humoral immunity in chickens and found even a lower dose of this compound could enhance the development of the immune organs, increase leukocyte count and the ratio of lymphocytes, and contribute in the rise of antibody titers against NDV. PFFPS increased the concentrations of IL-2 and IFIV-gamma, along with the NDV specific secretory IgA levels (SIgA) in the duodenum.

Lignin showed a positive response in birds while fed with 0.5% purified lignin in the blood lymphocyte population [121]. Hussein et al. [122] looked at lignocellulose supplementation in layer pullets with three diets at the age of 4 weeks; control commercial diet, control plus 1% insoluble fiber, and control plus 1% mixed soluble/insoluble fiber. After 4 weeks, birds fed either of the supplemented diets had larger thymus gland and bursa of Fabricius, and they had more Peyer’s patches than the unsupplemented control group. At 14 weeks of age, the pullets fed insoluble fiber supplemented diets had more heterophil phagocytosis activity, indicating increased innate immune functions. Hussein and Frankel [123] also found birds fed with a lignin-containing diet had, on the average larger bursa of Fabricius and more Peyer’s patches. Birds fed with a fructooligosaccharide supplemented diet also showed positive responses towards the enhancement of innate and adaptive immunity in chicken. Shang et al. [124] fed 0.5% fructooligosaccharide supplemented diet in *Salmonella* challenged broilers, which decreased heterophil activity, but monocyte activity and serum IgY concentration increased while and interferon-γ and IL-10 were upregulated in the ileum. The phenolic units of purified lignin exert antimicrobial function, and lignin improves the production and health of broilers. Lignin also increases CD4^+^ and CD8^+^ lymphocytes in the duodenum [123].

Fructans are related to the immune responses of gut-associated lymphoid tissue (GALT) and the systemic immune system. Fructans act as an immunomodulator in three different ways. Firstly, by increasing the relative abundance of *Bifidobacteria, fructans* stimulate the secretion of cytokines or antibodies. Secondly, SCFA produced from fructans could activate leukocytes. Thirdly, the carbohydrate receptors on the surface of immune cells could recognize fructans [125].

## Effects of dietary fiber on the environment

Ammonia emission is a major concern in the poultry industry; part of the excreted N is volatilized as NH_3_ and spread to the atmosphere. In the United States, the total amount of NH_3_ emission from animal husbandry activities was 80.9%, while 26.7% were only from poultry [126, 127]. Several experiments have proven that NH_3_ emission has a bad influence on poultry’s health and performance. It impairs macrophage function, reduces lung function, results in a lower BWG, and increases susceptibility to diseases like Newcastle disease, which may cause blindness [128]. The poor health condition of poultry also leads to lower egg production. Ammonia emission from poultry farms even impacts the environment by eutrophication of surface water resources and nuisance odors [129]. DF supplementation may have a modifying role on the enterohepatic cycle of N and amnion acid loss. However, Roberts et al. [59] reported no adverse effect on egg production, BWG, and FCR when increasing the DF and decreasing the crude protein content in laying hens’ diets. Thus, DF’s inclusion in the hen’s diet may be a feasible option to mitigate NH_3_ emission by increasing N consumption in a commercial egg-production operation.

## Adverse effects of dietary fiber in poultry

Feeding fiber to poultry has generally been discouraged, primarily because of the adverse effects that fiber exerts on performance and nutrient utilization. As mentioned above, some components in plants cannot benefit the GIT of poultry. Cellulose and hemicellulose are not well digested. Arabinoxylans and pentosans present in cereals like rye result in a low nutritional value. The high concentration of β-glucans in barley is responsible for its low nutritional value. Also, the inclusion of high fiber ingredients is usually limited because of the poor metabolizable energy contents [4]. Several experiments with alfalfa indicated that saponins in it might prevent hypercholesterolemia, reduce egg production, and depress growth in mammals and birds [19]. Soluble fiber pectin increased digesta viscosity and exhibited antinutritive effects in the young chick with decreased growth performance [82]. Besides, raffinose is a type of oligosaccharides that vary in distribution among leguminous species and varieties of the same species. Because it cannot be digested in the upper digestive tract, it remains undigested until it reaches the lower gut, where it can be fermented by microbial enzymes. A byproduct of the fermentation of these compounds is the production of gases that have been associated with flatulence in non-ruminant animals, as well as causing diarrhea [130].

Supplementing such diets with specific feed enzymes targeting these compounds may improve the nutritional value of fiber. A recent study from our lab has shown that a combination of xylanase, amylase, and protease supplements added in corn-SBM based diet of broilers could optimize the utilization of fiber for better average daily gain and improved feed efficiency while maintaining performance to a level comparable to that of the costly conventional feedstuff-based diets with low fiber content [131].

## Conclusion

Although DF was considered an antinutritional factor in the past, there is increasing interest in their use in poultry nutrition due to claimed benefits. The inclusion of an appropriate amount of DF in poultry diets promotes gastrointestinal tract development and improves nutrient utilization, growth performance, and gut health parameters (as summarized in Table 1), including beneficial microbiota, immune system, and avoids overconsumption without hindering the growth of poultry. Difference sources, type, and form of DF have been used in poultry both *in vivo* and *in ovo* to evaluate various parameters in poultry with some confirming results along with some contradictory reports of not getting the benefits all time. However, it is necessary to determine the type, form, and inclusion level of fiber in poultry diets to attain optimal performance and economic benefits under commercial conditions. Furthermore, it would be desirable to formulate diets with the amount of fiber to satisfy the needs of poultry in different stages of growth and production. However, a gap in knowledge remains in the underlying mechanism of how different types of fibers affect the gut health of poultry. Also, it is worth studying the effect of fiber inclusion in poultry diets from an economic and environmental point of view, which will have practical significance.

**Table 1:** Effect of dietary fibers on gut health of poultry

Source	Inclusion level	Species	Response	Reference
SBM/Oat hull	3%, 5%	Broiler	Increased gizzard weight and reduced it’s pH	[4]
SBP/Pea hull	7.5%	Broiler	Increased abrasion of the mucosal surface of the small intestine, shortened the villus, and increased mucus output	[24]
Pea hull	2.5%	Broiler	Increased VH:CD ratio	[24]
SFM	40–45 g/kg	Pullet	Increased VH, villus weight, the VH:CD ratio and surface area	[25]
SFM	47 g/kg	Layer	Increased VH, villus weight, the VH:CD ratio and surface area	[26]
SFM	97 g/kg	Layer	Decreased VH, villus weight, the VH:CD ratio and surface area	[26]
Soyabean hull	40 g/Kg	Broiler	Decreased negative effects of coccidiosis on VH:CD	[55]
Rice hull	40 g/Kg	Broiler	Decreased negative effects of coccidiosis on duodenal villus height	[55]
Wheat bran	30 g/Kg	Broiler	Increased VH and VH:CD ratio	[56]
Chitooligosaccharide	5 mg/egg (*in ovo* injection)	Broiler	Increased beneficial gut bacteria	[62]
Soybean hull	7%	Broiler	increased acetic, propionic, isobutyric and butyric acid, lactic and succinic acid in cecal contents and depressed formic acid production	[76]
Alfalfa extract	0.06%	Broiler	Increased thymus, spleen and bursa weight; Proliferation of T and B lymphocytes; Increased anti-NDV serum; Enhanced overall immunity	[19]
Algae-derived polysaccharides	2500 mg/kg	Broiler	Increased VH of jejunum and ileum	[85]
Algae derived polysaccharides	4000 mg/kg	Broiler	Increased the VH of duodenum and ileum	[85]
Yeast derived β-glucans	0, 0.02%, or 0.1%	Broiler	Altered the cytokine-chemokine balance	[86]
Raffinose	1.5, 3.0, and 4.5 mg in 0.2 mL of an aqueous diluents	Broiler	Increased VH and VH:CH ratio; improved immunity and gut health	[92]
Yeast cell	2.5 g/kg	Broiler	Enhanced antibody titers, IgM and IgA concentration, serum lysozyme and better gut morphology	[97]
Yeast culture	20 g/t	Turkey	Reduced transport stress	[103]
Wheat based diet (arabinoxylans)	0.1% (wt/wt)	Broiler	Increased production of cecal SCFA	[106]
Arabinoxylooligosaccharides	0.4% or 0.2%	Broiler	Maintained gut health	[107]
Barley-derived arabinoxylans	200 mg/kg body weight or 300 mg/kg body weight	Broiler	Enhanced macrophage activity	[109]
Fructooligosaccharide	0.5%	Broiler	Upregulated interferon-γ and IL-10 expression in ileum	[124]

*Abbreviations: SBM* soybean meal, *SBP* sugar beet pulp, *SCFA* short chain fatty acid, *SFM* Sunflower meal, *VH* villus height, *VH:CD* villus height to crypt depth ratio

## Data Availability

None.

## Abbreviations

AGP: Antibiotic growth promoter
APS: Astragalus polysaccharide
BWG: Body weight gain
CD: Crypt depth
COS: Chitosan oligosaccharide
d: Day
DF: Dietary fiber
DM: Dry matter
FCR: Feed conversion ratio
FI: Feed Intake
GALT: Gut-associated lymphoid tissue
GIT: Gastrointestinal tract
GOS: Galacto oligosaccharide
HCL: Hydrochloric acid
IBD: Infectious bursal disease
IBV: Infectious bronchitis virus
IgA: Immunoglobulin A
IgG: Immunoglobulin G
IgM: Immunoglobulin M
IgY: Immunoglobulin Y
IL: Interleukin
LPS: Lipopolysaccharide
mRNA: Messenger ribonucleic acid
NDV: Newcastle disease virus
NSP: Non-search polysaccharides
PFFPS: *Paulownia fortunei* flower polysaccharide
PMA: Phorbol-12-myristate-13-acetate
SBM: Soyabean meal
SBP: Sugar beet pulp
SCFA: Short chain fatty acid
SFH: Sunflower hull
VH: Villus height
WGS: Whole genome sequencing

## References

[1] Hipsley EH (1953). Dietary "fibre" and pregnancy toxaemia. Br Med J.

[2] Bach Knudsen KE (2001). The nutritional significance of “dietary fibre” analysis. Anim Feed Sci Technol.

[3] Jha R, Berrocoso JD (2015). Review: dietary fiber utilization and its effects on physiological functions and gut health of swine. Animal..

[4] Jiménez-Moreno E, Mateos GG. Use of dietery fiber in broilers. San Juan del Rio, Queretaro: In Memorias De La Sexta Reunión Anual Aecacem 2013; 2013.

[5] González-Alvarado JM, Jiménez-Moreno E, González-Sánchez D, Lázaro R, Mateos GG (2010). Effect of inclusion of oat hulls and sugar beet pulp in the diet on productive performance and digestive traits of broilers from 1 to 42 days of age. Anim Feed Sci Technol.

[6] Jha R, Woyengo TA, Li J, Bedford MR, Vasanthan T, Zijlstra RT (2015). Enzymes enhance degradation of the fiber-starch-protein matrix of distillers dried grains with solubles as revealed by a porcine *in vitro* fermentation model and microscopy. J Anim Sci.

[7] Mateos GG, Jiménez-Moreno E, Serrano MP, Lázaro RP (2012). Poultry response to high levels of dietary fiber sources varying in physical and chemical characteristics. J Appl Poult Res.

[8] Jha R, Zijlstra RT. Physico-chemical properties of purified fiber affect their *in vitro* fermentation characteristics and are linked to *in vivo* characteristics in pigs. Can J Anim Sci. 2018;98(2):394–8.

[9] Williams BA, Grant LJ, Gidley MJ, Mikkelsen D (2017). Gut fermentation of dietary fibres: physico-chemistry of plant cell walls and implications for health. Int J Mol Sci.

[10] Jørgensen H, Zhao XQ, Knudsen KE, Eggum BO (1996). The influence of dietary fibre source and level on the development of the gastrointestinal tract, digestibility and energy metabolism in broiler chickens. Br J Nutr.

[11] Hartini S, Choct M, Hinch G, Kocher A, Nolan JV (2002). Effects of light intensity during rearing and beak trimming and dietary fiber sources on mortality, egg production, and performance of ISA brown laying hens. J Appl Poult Res.

[12] Jha R, Fouhse JM, Tiwari UP, Li L, Willing BP (2019). Dietary fiber and intestinal health of monogastric animals. Front Vet Sci..

[13] Adhikari P, Kiess A, Adhikari R, Jha R (2020). An approach to alternative strategies to control avian coccidiosis and necrotic enteritis. J Appl Poult Res.

[14] Vierira SL, Penz AM, Leboute EM, Corteline J (1992). A nutritional evaluation of a high fiber sunflower meal. J Appl Poult Res.

[15] Pettersson D, Razdan A (1993). Effects of increasing levels of sugar-beet pulp in broiler chicken diets on nutrient digestion and serum lipids. Br J Nutr.

[16] Nagalakshmi D, Rao SVR, Panda AK, Sastry VR (2007). Cottonseed meal in poultry diets: a review. J Poult Sci.

[17] Jha R, Leterme P (2012). Feed ingredients differing in fermentable fibre and indigestible protein content affect fermentation metabolites and faecal nitrogen excretion in growing pigs. Animal..

[18] Donalson LM, Kim WK, Woodward CL, Herrera P, Kubena LF, Nisbet DJ (2005). Utilizing different ratios of alfalfa and layer ration for molt induction and performance in commercial laying hens. Poult Sci.

[19] Dong XF, Gao WW, Tong JM, Jia HQ, Sa RN, Zhang Q (2007). Effect of polysavone (alfalfa extract) on abdominal fat deposition and immunity in broiler chickens. Poult Sci.

[20] Jha R, Singh AK, Yadav S, Berrocoso JFD, Mishra B (2019). Early nutrition programming (*in ovo* and post-hatch feeding) as a strategy to modulate gut health of poultry. Front Vet Sci.

[21] Hetland H, Choct M, Svihus B (2004). Role of insoluble non-starch polysaccharides in poultry nutrition. Worlds Poult Sci J.

[22] Celi P, Cowieson AJ, Fru-Nji F, Steinert RE, Kluenter AM, Verlhac V (2017). Gastrointestinal functionality in animal nutrition and health: new opportunities for sustainable animal production. Anim Feed Sci Technol.

[23] Kimiaeitalab MV, Mirzaie Goudarzi S, Jiménez-Moreno E, Cámara L, Mateos GG (2018). A comparative study on the effects of dietary sunflower hulls on growth performance and digestive tract traits of broilers and pullets fed a pullet diet from 0 to 21days of age. Anim Feed Sci Technol.

[24] Jiménez-Moreno E, Romero C, Berrocoso J, Frikha M, Gonzalez-Mateos G. Effects of the inclusion of oat hulls or sugar beet pulp in the diet on gizzard characteristics, apparent ileal digestibility of nutrients, and microbial count in the ceca in 36 day old broilers reared on floor. St. Louis, EEUU: In 100 th Annual Meeting Poultry Science Association; 2011.

[25] Tüzün AE, Koçer B, Ege G, Bozkurt M. Influence of sunflower meal utilisation on growth performance and digestive tract traits of white strain pullets fed from 29 to 112 d of age. Br Poult Sci. 2020:1–8. 10.1080/00071668.2020.1851353 33196293

[26] Koçer B, Bozkurt M, Ege G, Tüzün AE (2021). Effects of sunflower meal supplementation in the diet on productive performance, egg quality and gastrointestinal tract traits of laying hens. Br Poult Sci.

[27] Hsiao HY, Anderson DM, Dale NM (2006). Levels of β-mannan in soybean meal. Poult Sci.

[28] Zijlstra RT, Jha R, Woodward AD, Fouhse J, van Kempen TATG. Starch and fiber properties affect their kinetics of digestion and thereby digestive physiology in pigs. J Anim Sci. 2012;90(suppl_4):49–58. 10.2527/jas.53718 23365281

[29] Slama J, Schedle K, Wurzer GK, Gierus M (2019). Physicochemical properties to support fibre characterization in monogastric animal nutrition. J Sci Food Agric.

[30] Sadeghi A, Toghyani M, Gheisari A (2015). Effect of various fiber types and choice feeding of fiber on performance, gut development, humoral immunity, and fiber preference in broiler chicks. Poult Sci.

[31] Hosseindoust A, Lee S, Gook Nho W, Song YH, Shin JS, Laxman Ingale S (2019). A dose–response study to evaluate the effects of pH-stable β-mannanase derived from *Trichoderma citrinoviride* on growth performance, nutrient retention, and intestine morphology in broiler chickens. Ital J Anim Sci.

[32] Pedersen NR, Ravn JL, Pettersson D. A multienzyme NSP product solubilises and degrades NSP structures in canola and mediates protein solubilisation and degradation *in vitro*. Anim Feed Sci Technol. 2017;234:244–52.

[33] Singh AK, Tiwari UP, Berrocoso JD, Dersjant-Li Y, Awati A, Jha R (2019). Effects of a combination of xylanase, amylase and protease, and probiotics on major nutrients including amino acids and non-starch polysaccharides utilization in broilers fed different level of fibers. Poult Sci.

[34] Li YP, Wang ZY, Yang HM, Xu L, Xie YJ, Jin SL (2017). Effects of dietary fiber on growth performance, slaughter performance, serum biochemical parameters, and nutrient utilization in geese. Poult Sci.

[35] Li Y, Yang H, Xu L, Wang Z, Zhao Y, Chen X (2018). Effects of dietary fiber levels on cecal microbiota composition in geese. Asian-Australas J Anim Sci.

[36] Svihus B, Juvik E, Hetland H, Krogdahl Å (2004). Causes for improvement in nutritive value of broiler chicken diets with whole wheat instead of ground wheat. Br Poult Sci.

[37] Svihus B (2011). The gizzard: function, influence of diet structure and effects on nutrient availability. Worlds Poult Sci J..

[38] Abdollahi MR, Zaefarian F, Hunt H, Anwar MN, Thomas DG, Ravindran V (2019). Wheat particle size, insoluble fibre sources and whole wheat feeding influence gizzard musculature and nutrient utilisation to different extents in broiler chickens. J Anim Physiol Anim Nutr.

[39] Hetland H, Svihus B, Choct M (2005). Role of insoluble fiber on gizzard activity in layers. J Appl Poult Res.

[40] Duke G. Alimentary canal: Secretions and digestion, special digestion functions and absorption. In: Sturkie P, editor. Avian Physiology. New York; Springer-Verlag; 1986. p. 289–302.

[41] Guinotte F, Gautron J, Nys Y, Soumarmon A (1995). Calcium solubilization and retention in the gastrointestinal tract in chicks (*Gallus domesticus*) as a function of gastric acid secretion inhibition and of calcium carbonate particle size. Br J Nutr.

[42] Mateos GG, Lázaro R, Gracia MI (2002). The feasibility of using nutritional modifications to replace drugs in poultry feeds. J Appl Poult Res.

[43] González-Alvarado JM, Jiménez-Moreno E, Lázaro R, Mateos GG (2007). Effect of type of cereal, heat processing of the cereal, and inclusion of fiber in the diet on productive performance and digestive traits of broilers. Poult Sci.

[44] Jiménez-Moreno E, González-Alvarado JM, de Coca-Sinova A, Lázaro R, Mateos GG (2009). Effects of source of fibre on the development and pH of the gastrointestinal tract of broilers. Anim Feed Sci Technol.

[45] Mateos GG, Martín F, Latorre MA, Vicente B, Lázaro R (2006). Inclusion of oat hulls in diets for young pigs based on cooked maize or cooked rice. Anim Sci.

[46] Ling J. Gao YY, Hui Y, Wang WC, Lin ZP, Yang HY, et al. Effects of dietary fiber and grit on performance, gastrointestinal tract development, lipometabolism, and grit retention of goslings. J Integr Agric. 2014;13(12):2731–40.

[47] Kalmendal R, Elwinger K, Holm L, Tauson R (2011). High-fibre sunflower cake affects small intestinal digestion and health in broiler chickens. Bri Poult Sci.

[48] Amerah AM, Ravindran V, Lentle RG (2009). Influence of insoluble fibre and whole wheat inclusion on the performance, digestive tract development and ileal microbiota profile of broiler chickens. Br Poult Sci.

[49] Houshmand M, Azhar K, Zulkifli I, Bejo MH, Meimandipour A, Kamyab A (2011). Effects of non-antibiotic feed additives on performance, tibial dyschondroplasia incidence and tibia characteristics of broilers fed low-calcium diets. J Anim Physiol Anim Nutr.

[50] Tako E, Glahn RP, Knez M, Stangoulis JC (2014). The effect of wheat prebiotics on the gut bacterial population and iron status of iron deficient broiler chickens. Nutr J.

[51] Loddi MM, Moraes VM, Nakaghi LSO, Tucci FM, Hannas MI, Ariki JA. Mannan oligosaccharide and organic acids on performance and intestinal morphometric characteristics of broiler chickens. New York, USA: In Proceedings of the 20th Annual Symposium on Computational Geometry; 2004.

[52] Maiorka A, Santin A, Borges S, Opalinski M, Silva A (2004). Evaluation of a mix of fumaric, lactic, citric and ascorbic acids on start diets of broilers. Arch Vet Sci.

[53] Loddi M. Probioticos and prebioticos e acidificanate organico. Taboticabal. FCAV, UNE SP: In em dietas para frangos de corte [tese]; 2003.

[54] Pelicano E, Souza P, Souza H, Figueiredo D, Boiago M, Carvalho S (2005). Intestinal mucosa development in broiler chicken fed natural growth promoters. Braz J Poult Sci.

[55] Sadeghi A, Toghyani M, Tabeidian SA, Foroozandeh AD, Ghalamkari G (2020). Efficacy of dietary supplemental insoluble fibrous materials in ameliorating adverse effects of coccidial challenge in broiler chickens. Arch Anim Nutr.

[56] Shang Q, Wu D, Liu H, Mahfuz S, Piao X (2020). The impact of wheat bran on the morphology and physiology of the gastrointestinal tract in broiler chickens. Animals..

[57] Walugembe M, Rothschild MF, Persia ME (2014). Effects of high fiber ingredients on the performance, metabolizable energy and fiber digestibility of broiler and layer chicks. Anim Feed Sci Technol.

[58] Saadatmand N, Toghyani M, Gheisari A (2019). Effects of dietary fiber and threonine on performance, intestinal morphology and immune responses in broiler chickens. Anim Nutr.

[59] Roberts SA, Xin H, Kerr BJ, Russell JR, Bregendahl K (2007). Effects of dietary fiber and reduced crude protein on nitrogen balance and egg production in laying hens. Poult Sci.

[60] Yadav S, Jha R (2019). Strategies to modulate the intestinal microbiota and their effects on nutrient utilization, performance, and health of poultry. J Anim Sci Biotechnol.

[61] Choct M (2009). Managing gut health through nutrition. Br Poult Sci.

[62] Zhang J, Cai K, Mishra R, Jha R (2020). *In ovo* supplementation of chitooligosaccharide and chlorella polysaccharide affects cecal microbial community, metabolic pathways, and fermentation metabolites in broiler chickens. Poult Sci.

[63] Gong J, Si W, Forster RJ, Huang R, Yu H, Yin Y (2007). 16S rRNA gene-based analysis of mucosa-associated bacterial community and phylogeny in the chicken gastrointestinal tracts: from crops to ceca. FEMS Microbiol Ecol.

[64] Wei S, Morrison M, Yu Z (2013). Bacterial census of poultry intestinal microbiome. Poult Sci.

[65] Jha R, Das R, Oak S, Mishra P (2020). Probiotics (direct-fed microbials) in poultry nutrition and their effects on nutrient utilization, growth and laying performance, and gut health: a systematic review. Animals..

[66] Liu B, Wang W, Zhu X, Sun X, Xiao J, Li D (2018). Response of gut microbiota to dietary fiber and metabolic interaction with SCFAs in piglets. Front Microbiol.

[67] Dunislawska A, Slawinska A, Stadnicka K, Bednarczyk M, Gulewicz P, Jozefiak D (2017). Synbiotics for broiler chickens-*in vitro* design and evaluation of the influence on host and selected microbiota populations following *in ovo* delivery. PLoS One.

[68] CK Rajendran SR, Okolie CL, Udenigwe CC, Mason B. (2017). Structural features underlying prebiotic activity of conventional and potential prebiotic oligosaccharides in food and health. J Food Biochem.

[69] Jiménez-Moreno E, de Coca-Sinova A, González-Alvarado JM, Mateos GG (2016). Inclusion of insoluble fiber sources in mash or pellet diets for young broilers. 1. Effects on growth performance and water intake. Poult Sci.

[70] Bedbury HP, Duke GE (1983). Cecal microflora of turkeys fed low or high fiber diets: enumeration, identification, and determination of cellulolytic activity. Poult Sci.

[71] Xu ZR, Hu CH, Xia MS, Zhan XA, Wang MQ (2003). Effects of dietary fructooligosaccharide on digestive enzyme activities, intestinal microflora and morphology of male broilers. Poult Sci.

[72] Chen WL, Liang JB, Jahromi MF, Abdullah N, Ho YW, Tufarelli V (2015). Enzyme treatment enhances release of prebiotic oligosaccharides from palm kernel expeller. BioResources..

[73] Abazari A, Navidshad B, Mirzaei Aghjehgheshlagh F, Nikbin S (2016). The effect of rice husk as an insoluble dietary fiber source on intestinal morphology and *Lactobacilli* and *Escherichia coli* populations in broilers. Iran J Vet REes.

[74] Liu G, Luo X, Zhao X, Zhang A, Jiang N, Yang L (2018). Gut microbiota correlates with fiber and apparent nutrients digestion in goose. Poult Sci.

[75] Montagne L, Pluske JR, Hampson DJ (2003). A review of interactions between dietary fibre and the intestinal mucosa, and their consequences on digestive health in young non-ruminant animals. Anim Feed Sci Technol.

[76] Wu SB, Mikkelsen LL, Torok VA, Iji PA, Setia S, Hughes RJ. Role of dietary fibre and litter type on development of necrotic enteritis in broiler chickens challenged with *Clostridium perfringens*. Sydney, Australia: In Proceedings of the Australian Poultry Science Symposium; 2011.

[77] Walugembe M, Hsieh JC, Koszewski NJ, Lamont SJ, Persia ME, Rothschild MF (2015). Effects of dietary fiber on cecal short-chain fatty acid and cecal microbiota of broiler and laying-hen chicks. Poult Sci.

[78] Shakouri MD, Iji PA, Mikkelsen LL, Cowieson AJ (2009). Intestinal function and gut microflora of broiler chickens as influenced by cereal grains and microbial enzyme supplementation. J Anim Physiol Anim Nutr.

[79] Akbaryan M, Mahdavi A, Jebelli-Javan A, Staji H, Darabighane B (2019). A comparison of the effects of resistant starch, fructooligosaccharide, and zinc bacitracin on cecal short-chain fatty acids, cecal microflora, intestinal morphology, and antibody titer against Newcastle disease virus in broilers. Comp Clin Path.

[80] Johnson TJ, Shank JM, Johnson JG (2017). Current and potential treatments for reducing *Campylobacter* colonization in animal hosts and disease in humans. Front Microbiol.

[81] Baurhoo B, Phillip L, Ruiz-Feria CA (2007). Effects of purified lignin and mannan oligosaccharides on intestinal integrity and microbial populations in the ceca and litter of broiler chickens. Poult Sci.

[82] Wils-Plotz EL, Jenkins MC, Dilger RN (2013). Modulation of the intestinal environment, innate immune response, and barrier function by dietary threonine and purified fiber during a coccidiosis challenge in broiler chicks. Poult Sci.

[83] Kim WK, Lillehoj HS (2019). Immunity, immunomodulation, and antibiotic alternatives to maximize the genetic potential of poultry for growth and disease response. Anim Feed Sci Technol.

[84] Klasing KC (2007). Nutrition and the immune system. Bri Poult Sci..

[85] Liu WC, Guo Y, Zhao ZH, Jha R, Balasubramanian B (2020). Algae-derived polysaccharides promote growth performance by improving antioxidant capacity and intestinal barrier function in broiler chickens. Front Vet Sci..

[86] Cox CM, Stuard LH, Kim S, McElroy AP, Bedford MR, Dalloul RA (2010). Performance and immune responses to dietary beta-glucan in broiler chicks. Poult Sci.

[87] Cox CM, Sumners LH, Kim S, McElroy AP, Bedford MR, Dalloul RA (2010). Immune responses to dietary beta-glucan in broiler chicks during an *Eimeria* challenge. Poult Sci.

[88] Tian X, Shao Y, Wang Z, Guo Y (2016). Effects of dietary yeast β-glucans supplementation on growth performance, gut morphology, intestinal *Clostridium perfringens* population and immune response of broiler chickens challenged with necrotic enteritis. Anim Feed Sci Technol.

[89] Ott CP, Omara II, Persia ME, Dalloul RA (2018). The impact of β-glucans on performance and response of broiler chickens during a coccidiosis challenge. Poult Sci.

[90] Shao Y, Guo Y, Wang Z (2013). β-1,3/1,6-Glucan alleviated intestinal mucosal barrier impairment of broiler chickens challenged with *Salmonella enterica* serovar Typhimurium. Poult Sci.

[91] Horst G, Levine R, Chick R, Hofacre C (2019). Effects of beta-1,3-glucan (AletaTM) on vaccination response in broiler chickens. Poult Sci.

[92] Berrocoso JD, Kida R, Singh AK, Kim YS, Jha R (2017). Effect of *in ovo* injection of raffinose on growth performance and gut health parameters of broiler chicken. Poult Sci.

[93] Zheng L, Kelly CJ, Battista KD, Schaefer R, Lanis JM, Alexeev EE (2017). Microbial-derived butyrate promotes epithelial barrier function through IL-10 receptor-dependent repression of claudin-2. J Immunol.

[94] Zhou ZY, Packialakshmi B, Makkar SK, Dridi S, Rath NC (2014). Effect of butyrate on immune response of a chicken macrophage cell line. Vet Immunol Immunopathol.

[95] Wrzosek L, Miquel S, Noordine ML, Bouet S, Joncquel Chevalier-Curt M, Robert V (2013). *Bacteroides thetaiotaomicron* and *Faecalibacterium prausnitzii* influence the production of mucus glycans and the development of goblet cells in the colonic epithelium of a gnotobiotic model rodent. BMC Biol.

[96] Ahiwe EU, Chang'a EP, Abdallh ME, Al-Qahtani M, Kheravii SK, Wu S (2019). Dietary hydrolysed yeast cell wall extract is comparable to antibiotics in the control of subclinical necrotic enteritis in broiler chickens. Br Poult Sci.

[97] Gao J, Zhang HJ, Yu SH, Wu SG, Yoon I, Quigley J (2008). Effects of yeast culture in broiler diets on performance and immunomodulatory functions. Poul Sci.

[98] Alizadeh M, Rodriguez-Lecompte JC, Yitbarek A, Sharif S, Crow G, Slominski BA (2016). Effect of yeast-derived products on systemic innate immune response of broiler chickens following a lipopolysaccharide challenge. Poult Sci.

[99] Munyaka PM, Echeverry H, Yitbarek A, Camelo-Jaimes G, Sharif S, Guenter W (2012). Local and systemic innate immunity in broiler chickens supplemented with yeast-derived carbohydrates. Poult Sci.

[100] Li Z, Wang W, Lv Z, Liu D, Guo Y (2017). *Bacillus subtilis* and yeast cell wall improve the intestinal health of broilers challenged by *Clostridium perfringens*. Br Poult Sci.

[101] Awadin WF, Eladl AH, El-Shafei RA, El-Adl MA, Ali HS (2019). Immunological and pathological effects of vitamin E with Fetomune plus(®) on chickens experimentally infected with avian influenza virus H9N2. Vet Microbiol.

[102] Huff GR, Dutta V, Huff WE, Rath NC (2011). Effects of dietary yeast extract on turkey stress response and heterophil oxidative burst activity. Br Poult Sci.

[103] Huff GR, Huff WE, Jalukar S, Oppy J, Rath NC, Packialakshmi B (2013). The effects of yeast feed supplementation on Turkey performance and pathogen colonization in a transport stress/*Escherichia coli* challenge. Poult Sci.

[104] Tiwari UP, Singh AK, Jha R (2019). Fermentation characteristics of resistant starch, arabinoxylan, and β-glucan and their effects on the gut microbial ecology of pigs: a review. Anim Nutr..

[105] Tiwari UP, Chen H, Kim SW, Jha R (2018). Supplemental effect of xylanase and mannanase on nutrient digestibility and gut health of nursery pigs studied using both *in vivo* and *in vitro* models. Anim Feed Sci Technol.

[106] Yacoubi N, Saulnier L, Bonnin E, Devillard E, Eeckhaut V, Rhayat L (2018). Short-chain arabinoxylans prepared from enzymatically treated wheat grain exert prebiotic effects during the broiler starter period. Poult Sci.

[107] Tiwari UP, Fleming SA, Abdul Rasheed MS, Jha R, Dilger RN (2020). The role of oligosaccharides and polysaccharides of xylan and mannan in gut health of monogastric animals. J Nutr Sci.

[108] Eeckhaut V, Van Immerseel F, Dewulf J, Pasmans F, Haesebrouck F, Ducatelle R (2008). Arabinoxylooligosaccharides from wheat bran inhibit *Salmonella* colonization in broiler chickens. Poult Sci.

[109] Akhtar M, Humayun M, Iqbal Z, Awais MM, Anwar MI (2018). Evaluation of biological response modification and immunotherapeutic activities of barley-derived arabinoxylans against coccidiosis in commercial broilers. Turk J Vet Anim Sci.

[110] Huang R-L, Deng Z-Y, Yang C-b, Yin Y-L, Xie MY, Wu G-Y (2007). Dietary oligochitosan supplementation enhances immune status of broilers. J Sci Food Agric.

[111] Park SH, Perrotta A, Hanning I, Diaz-Sanchez S, Pendleton S, Alm E (2017). Pasture flock chicken cecal microbiome responses to prebiotics and plum fiber feed amendments. Poult Sci.

[112] Biggs P, Parsons CM, Fahey GC (2007). The effects of several oligosaccharides on growth performance, nutrient digestibilities, and cecal microbial populations in young chicks. Poult Sci.

[113] Li SP, Zhao XJ, Wang JY (2009). Synergy of Astragalus polysaccharides and probiotics (*Lactobacillus* and *Bacillus cereus*) on immunity and intestinal microbiota in chicks. Poult Sci.

[114] Habijanic J, Berovic M, Boh B, Plankl M, Wraber B (2015). Submerged cultivation of *Ganoderma lucidum* and the effects of its polysaccharides on the production of human cytokines TNF-α, IL-12, IFN-γ, IL-2, IL-4, IL-10 and IL-17. New Biotechnol.

[115] Yan ZF, Liu NX, Mao XX, Li Y, Li CT (2014). Activation effects of polysaccharides of *Flammulina velutipes* mycorrhizae on the T lymphocyte immune function. J Immunol Res.

[116] Di H-Y, Zhang Y-Y, Chen D-F (2013). Isolation of an anti-complementary polysaccharide from the root of *Bupleurum chinense* and identification of its targets in complement activation cascade. Chin J Nat Med.

[117] Chen Y, Wang D, Hu Y, Guo Z, Wang J, Zhao X (2010). Astragalus polysaccharide and oxymatrine can synergistically improve the immune efficacy of Newcastle disease vaccine in chicken. Int J Biol Macromol.

[118] Shan C, Sun B, Dalloul RA, Zhai Z, Sun P, Li M (2019). Effect of the oral administration of *Astragalus* polysaccharides on jejunum mucosal immunity in chickens vaccinated against Newcastle disease. Microb Pathog.

[119] Guo FC, Kwakkel RP, Williams BA, Parmentier HK, Li WK, Yang ZQ (2004). Effects of mushroom and herb polysaccharides on cellular and humoral immune responses of *Eimeria tenella*-infected chickens. Poult Sci.

[120] Wang Q, Meng X, Zhu L, Xu Y, Cui W, He X (2019). A polysaccharide found in *Paulownia fortunei* flowers can enhance cellular and humoral immunity in chickens. Int J Biol Macromol.

[121] Slaminková Z, Revajová V, Levkut M, Leng L, Bořutová R (2009). Influence of deoxynivalenol and lignin on lymphocyte subpopulations in blood and intestinal mucosa in chickens. Folia Vet.

[122] Hussein SM, Yokhana JS, Frankel TL (2017). Supplementing the feeds of layer pullets, at different ages with two different fiber sources improves immune function. Poult Sci.

[123] Hussein SM, Frankel TL (2019). Effect of varying proportions of lignin and cellulose supplements on immune function and lymphoid organs of layer poultry (*Gallus gallus*). J Poult Sci.

[124] Shang Y, Regassa A, Kim JH, Kim WK (2015). The effect of dietary fructooligosaccharide supplementation on growth performance, intestinal morphology, and immune responses in broiler chickens challenged with *Salmonella *Enteritidis lipopolysaccharides. Poult Sci.

[125] Seifert S, Watzl B (2007). Inulin and oligofructose: review of experimental data on immune modulation. J Nutr.

[126] Li H, Xin H, Liang Y, Burns RT (2008). Reduction of ammonia emissions from stored laying hen manure through topical application of zeolite, Al+ clear, Ferix-3, or poultry litter treatment. J Appl Poult Res.

[127] Li H, Xin HJTotA. Lab-scale assessment of gaseous emissions from laying-hen manure storage as affected by physical and environmental factors. Trans ASABE 2010;53(2):593–604.

[128] Miles DM, Branton SL, Lott BD (2004). Atmospheric ammonia is detrimental to the performance of modern commercial broilers. Poult Sci.

[129] Ritz CW, Fairchild BD, Lacy MP (2004). Implications of ammonia production and emissions from commercial poultry facilities: a review. J Appl Poult Res.

[130] Jha R, Berrocoso JFD (2016). Dietary fiber and protein fermentation in the intestine of swine and their interactive effects on gut health and on the environment: a review. Anim Feed Sci Technol.

[131] Singh AK, Berrocoso JFD, Dersjant-Li Y, Awati A, Jha R (2017). Effect of a combination of xylanase, amylase and protease on growth performance of broilers fed low and high fiber diets. Anim Feed Sci Technol.

